# Euphorbiasteroid Induces Apoptosis as Well as Autophagy through Modulating SHP-1/STAT3 Pathway in Hepatocellular Carcinoma Cells

**DOI:** 10.3390/ijms241813713

**Published:** 2023-09-05

**Authors:** Na Young Kim, Gautam Sethi, Jae-Young Um, Kwang Seok Ahn

**Affiliations:** 1 Department of Science in Korean Medicine, Kyung Hee University, 24 Kyungheedae-ro, Dongdaemun-gu, Seoul 02447, Republic of Korea; nay0kim@naver.com (N.Y.K.); jyum@khu.ac.kr (J.-Y.U.); 2 Department of Pharmacology, Yong Loo Lin School of Medicine, National University of Singapore, Singapore 117600, Singapore; phcgs@nus.edu.sg

**Keywords:** euphorbiasteroid, STAT3, apoptosis, autophagy, SHP-1

## Abstract

Euphorbiasteroid (EPBS) has gained attention for its activity against human lung cancer and sarcoma; however, its impact on hepatocellular carcinoma has not yet been elucidated. Here, we investigated the cytotoxic effect of EPBS on human hepatocellular carcinoma (HCC) cells. We found that EPBS induced both apoptosis and autophagy in HCC cells. Additionally, we observed that EPBS treatment suppressed the constitutive as well as the inducible activation of a signal transducer and activator of transcription 3 (STAT3) protein expression. Moreover, EPBS promoted the expression of SHP-1 protein and the production of reactive oxidative stress (ROS). Furthermore, the knockdown of SHP-1 by siRNA transfection reversed the effects of EPBS, which have inductive effects related to apoptosis and autophagy. Therefore, EPBS can potentially function as an anti-cancer agent by inducing apoptosis and autophagy when targeting the SHP-1/STAT3 pathway.

## 1. Introduction

The abnormal expression of the signal transducer and activator of transcription 3 (STAT3) is observed in various malignancies, including breast cancer, pancreatic cancer, leukemia, and liver cancer [1,2,3,4,5]. This transcription factor is involved in various cell activities, such as growth, proliferation, metastasis, and survival [6,7]. STAT3 is phosphorylated by the protein kinases Janus kinase 1/2 (JAK1/2) and Src, and STAT3 phosphorylation is inhibited by protein phosphatases such as SHP-1, SHP-2, and the protein tyrosine phosphatase (PTP) epsilon [8,9]. High levels of p-STAT3 have been reported, especially in hepatocellular carcinoma [1]. The abnormal activity of the signal transducer and activator of transcription 3 (STAT3) can increase the expression of genes that affect cell growth (*Bcl-2*, *Bcl-xL*, *Survivin*, and *cyclin D1*) [10,11,12]. It has been reported that the high expression of STAT3 causes the occurrence of chemo-resistance and radio-resistance in human hepatocellular carcinoma (HCC) therapy; therefore, it is very important to discover anti-cancer agents that target STAT3 [1,7,13].

It has been reported that the inhibition of STAT3 activity could induce apoptosis and autophagy [14,15,16]. Apoptosis plays a key role in achieving cellular homeostasis and is characterized by chromatin condensation, cell shrinkage, and DNA fragmentation [17]. During apoptosis, the cleavage of poly (ADP-ribose) polymerase (PARP) is caused by caspase-3, and the expression of cleaved PARP serves as one of the representative apoptosis markers [18,19]. Also, autophagy is another cell death mechanism that maintains cell homeostasis. It plays a role in disassembling unnecessary or damaged proteins inside the cell and converting them into energy [20]. Autophagy acts as a double-edged sword in cancer. On one hand, it can act as a mechanism to supply nutrients to cancer cells, while on the other hand, excessive autophagy can cause cell death [21,22]. Anti-cancer drugs that can cause one type of cell death can lead to drug resistance. For this reason, drugs that can cause multiple types of cell death have emerged as important [23,24]. Apoptosis and autophagy can be induced by oxidative stress, and it is important to identify drugs that cause these two types of cell death at the same time.

Oxidative stress can have diverse effects on cancer cells [25]. For example, low amounts of reactive oxidative stress (ROS) usually help maintain cancer, but excessive amounts of ROS cause cell death [26]. ROS is also involved in the regulation of various proteins, especially transcription factor proteins such as STAT3 and nuclear factor kappa B (NF-κB) [27,28]. N-acetyl-l-cysteine (NAC) and glutathione (GSH) have the ability to inhibit and scavenge ROS [29,30]. It has been reported that NAC can prevent oxidative stress and promote the activity of antioxidant enzymes [31]. GSH is oxidized in the presence of ROS and converted to GSSG, which can prevent oxidative stress [32].

Euphorbiasteroid (EPBS) is not a steroid; rather, it is a lathyrane-type diterpene phytochemical component extracted from *Euphorbia lathyris* [33,34]. Based on a prior report by Kim [35], it can exhibit an anti-cancer effect by inhibiting the epidermal growth factor receptor (EGFR) and Wnt/β-catenin signaling in non-small cell lung cancer cells. Furthermore, EPBS can suppress the glycoprotein associated with multi-drug resistance in human sarcoma cells [36]. Additionally, Park et al. suggested that it could attenuate adipogenesis through the activation of the AMP-activated protein kinase (AMPK) pathway [34]. Since there are very few reports on the anti-cancer impact of EPBS, we investigated the influence of EPBS against HCC cells.

The number of HCC patients is increasing due to lifestyle and environmental factors [37,38]. The abnormal expression of STAT3 has been found in liver cancer, which is one of the causes of increased hepatocellular carcinoma progression [39,40]. Anti-cancer treatment with phytochemicals is very widely used with few side effects, especially as a mechanism that causes apoptosis [41,42]. However, because of the resistance to apoptosis induction in chemotherapy, drugs that induce cell death through autophagy induction have become important [23]. In this study, we examined the anti-cancer efficacy of EPBS in the HCC cell line. We also demonstrated that the inhibition of STAT3 activity by EPBS could effectively cause apoptosis and autophagy. The cellular activity of STAT3 was confirmed by the knockdown of the *SHP-1* gene, a protein phosphatase of p-STAT3, and the effect of EPBS was also confirmed. Based on our results, we suggest that EPBS can function as a potential anti-cancer molecule.

## 2. Results

### 2.1. EPBS Specifically Suppressed STAT3 Activation

**Figure 1:**
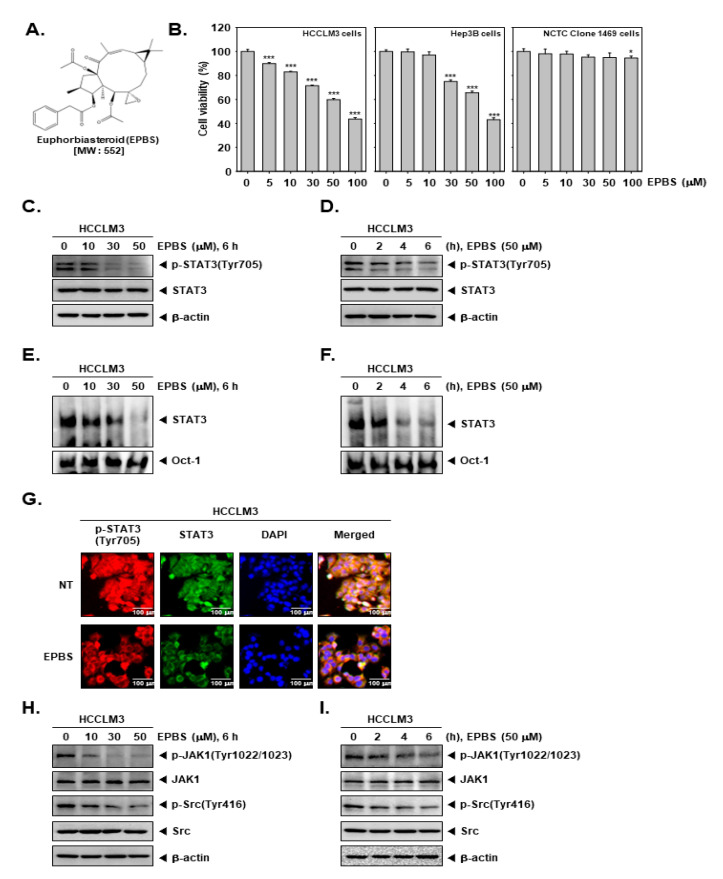
EPBS exhibited a cytotoxic effect and down–regulated the JAK/Src/STAT3 pathway. (**A**) The chemical structure of EPBS. (**B**) MTT assay was performed for measuring cell viability. HCCLM3 and Hep3B cells were treated with EPBS for 24 h. *** *p* < 0.001 and * *p* < 0.05 vs. non–treated (NT) cells. (**C**,**D**) HCCLM3 cells were treated with the indicated concentration of EPBS for the indicated time intervals. A Western blot analysis was performed to confirm protein levels. (**E**,**F**) HCCLM3 cells were treated with EPBS (0–10–30–50 µM or 50 µM) for 6 h or 0–2–4–6 h and an EMSA assay was performed. *Oct–1* was used for a control. (**G**) HCCLM3 cells were incubated with 0–50 µM of EPBS for 6 h and an ICC assay was conducted. DAPI staining was conducted to detect cell nuclei. (**H**,**I**) HCCLM3 cells were treated with 0–10–30–50 µM of EPBS for 6 h, or treated with 50 µM EPBS for 0–2–4–6 h. The cells were harvested, lysed, and then a Western blot analysis was thereafter conducted. Abbreviations: Euphorbiasteroid (EPBS); Janus kinase 1 (JAK1); signal transducer and activator of transcription 3 (STAT3).

The chemical structure of EPBS is shown in Figure 1A. We investigated whether EPBS can exhibit cytotoxicity against HCC cells. As demonstrated in Figure 1B, EPBS significantly suppressed the viability of the HCCLM3 and Hep3B cells. Since EPBS showed high cell toxicity (about 40% cell viability) at 100 µM, we set 50 µM as the maximum concentration for additional experiments. In a normal mouse liver NCTC clone 1469 cell line, EPBS treatment had little effect on cell viability. Thereafter, we examined the impact of EPBS on constitutive p-STAT3 expression by performing a Western blot analysis. The HCCLM3 cells were treated with EPBS in a concentration- (Figure 1C) and time-dependent manner (Figure 1D). The results suggested that EPBS could effectively inhibit the p-STAT3 (Tyr705), but that it has no impact on total STAT3.

### 2.2. EPBS Attenuated the Binding Ability of STAT3 and the Activation of STAT3 Upstream Kinases

We investigated the impact of EPBS on DNA binding ability by performing an EMSA assay. HCCLM3 cells were treated with different doses of EPBS (Figure 1E) for different time intervals (Figure 1F). The findings indicate that the DNA binding activity of STAT3 was attenuated after its exposure to EPBS. Additionally, we performed an ICC assay to observe the translocation ability of STAT3. The results depicted in Figure 1G suggest that the translocation of p-STAT3 into the nuclei was substantially inhibited after EPBS treatment. In Figure 1H,I, we observed that EPBS effectively suppressed the constitutive expression of p-JAK1 and p-Src at increased concentrations, or at various different time intervals.

### 2.3. EPBS Down-Modulated Inducible STAT3 Activation in Hep3B Cells

**Figure 2:**
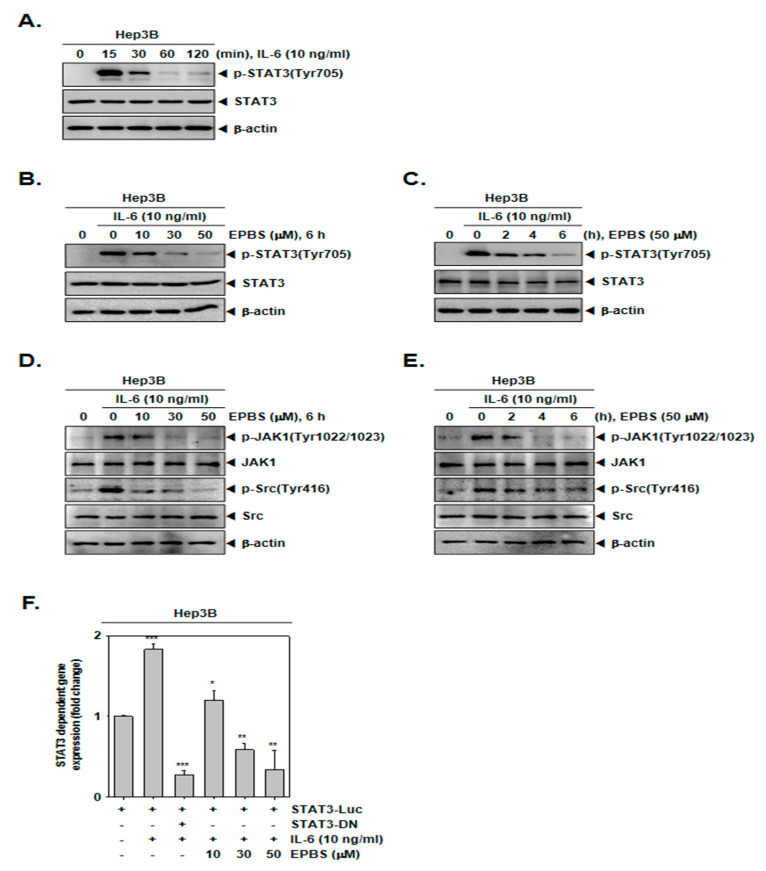
EPBS suppressed IL-6-stimulated STAT3 activation. (**A**) Hep3B cells were treated with 10 ng/mL of IL-6 for 0–15–30–60–120 min and Western blot analysis was performed. (**B**) Hep3B cells were pre–treated with the indicated concentration of EPBS for 6 h and then stimulated by 10 ng/mL IL-6 for 15 min, and Western blot analysis was conducted. (**C**) Hep3B cells were treated with EPBS (50 µM) for 0–2–4–6 h and then stimulated by IL-6 (10 ng/mL) for 15 min. The cells were harvested, lysed, and Western blot analysis was conducted. (**D**) The cells were treated with EPBS for 6 h and then treated with IL-6 for 15 min. (**E**) The cells were treated with EPBS and then IL-6 for the indicated time intervals. (**F**) Hep3B cells were transfected with STAT3 promoter luciferase for 24 h, then treated with EPBS (0–10–30–50 µM) for 6 h and stimulated with IL-6 for 15 min in a refreshed medium. STAT3-DN was used as a negative control. *** *p* < 0.001, ** *p* < 0.01, and * *p* < 0.05 vs. STAT3 promoter luciferase-transfected cells. Abbreviations: Euphorbiasteroid (EPBS); Interleukin-6 (IL-6); Janus kinase 1 (JAK1); signal transducer and activator of transcription 3 (STAT3).

Since Hep3B cells do not constitutively express p-STAT3, these cells were stimulated by IL-6 to achieve an inducible p-STAT3 [43]. First, we determined the time point at which the expression of the inducible p-STAT3 increased, and according to Figure 2A, we found that 15 min was the optimal time for IL-6 treatment. As depicted in Figure 2B,C, EPBS inhibited the IL-6-inducible STAT3 phosphorylation at each of the different dose levels, and for the different time intervals. In addition, it also reduced IL-6-induced JAK1 and Src kinase phosphorylation in Hep3B cells (Figure 2D,E).

### 2.4. EPBS Reduced STAT3 Gene Expression

Thereafter, we investigated whether EPBS can attenuate the activity of the STAT3 reporter gene. Hep3B cells were transfected with STAT3-luciferase DNA plasmid and treated with EPBS and IL-6. As shown in Figure 2F, the expression of the STAT3 reporter gene induced by IL-6 was substantially decreased following EPBS treatment in Hep3B cells.

### 2.5. Tyrosine Phosphatase Could Modulate p-STAT3 Inhibition by EPBS

**Figure 3:**
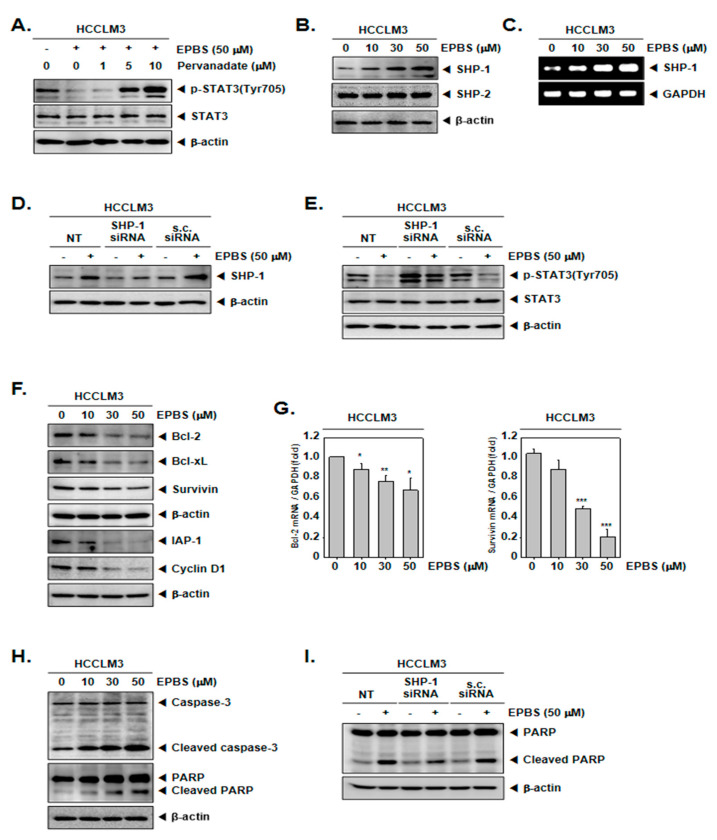
EPBS increased levels of various proteins. (**A**) HCCLM3 cells were co-treated with 50 µM of EPBS and 0–1–5–10 µM of pervanadate for 6 h. Thereafter, a Western blot analysis was conducted. (**B**,**C**) HCCLM3 cells were treated with EPBS (0–10–30–50 µM) for 6 h. Western blotting and RT-PCR were carried out. (**D**,**E**) HCCLM3 cells were transfected with *SHP-1* siRNA (50 nM) for 24 h and EPBS (50 µM) was treated for 6 h. Protein levels of SHP-1 and p-STAT3 were obtained by conducting a Western blot analysis. Scrambled siRNA (s.c. siRNA) was used for controls of transfection. (**F**,**G**) The cells were treated with 0–10–30–50 µM of EPBS for 24 h, and then collected. Western blot analysis was conducted to assess the expression level of the anti–apoptotic proteins, and real-time qPCR was performed to obtain the mRNA levels. *** *p* < 0.001, ** *p* < 0.01, and * *p* < 0.05 vs. non–treated (NT) cells. (**H**) EPBS was treated for 24 h and Western blotting was done. (**I**) HCCLM3 cells were transfected with *SHP-1* siRNA (50 nM) for 24 h and then treated with 50 µM of EPBS for 24 h. Western blot analysis was carried out. Abbreviations: Euphorbiasteroid (EPBS); Janus kinase 1 (JAK1); scrambled siRNA (s.c. siRNA); signal transducer and activator of transcription 3 (STAT3).

Because the inhibition of STAT3 phosphorylation could be induced by protein tyrosine phosphatase [44,45], we used sodium pervanadate, which is a phosphatase inhibitor. As indicated by the results in Figure 3A, the down-regulation of p-STAT3 by EPBS was reversed in the presence of pervanadate. These results showed that the EPBS-driven inhibition of p-STAT3 could be achieved using tyrosine phosphatase.

### 2.6. EPBS Upregulated SHP-1 in the Protein Levels and in mRNA Levels

As depicted in Figure 3B, the protein expression of SHP-1 increased upon treatment with EPBS in a dose-dependent fashion, but not that of the SHP-2 proteins. Furthermore, the mRNA levels of *SHP-1* concomitantly increased (Figure 3C). Furthermore, we investigated whether SHP-1 inhibition by siRNA transfection could attenuate the p-STAT3 inhibition by EPBS. As demonstrated in Figure 3D, the inhibition of SHP-1 proteins occurred successfully as a result of *SHP-1* siRNA transfection. Interestingly, EPBS-induced SHP-1 expression and the inhibition of p-STAT3 were clearly abrogated in *SHP-1* siRNA-transfected HCCLM3 cells (Figure 3E).

### 2.7. EPBS Abrogated the Oncogenic Proteins and Promoted Apoptotic Proteins

**Figure 4:**
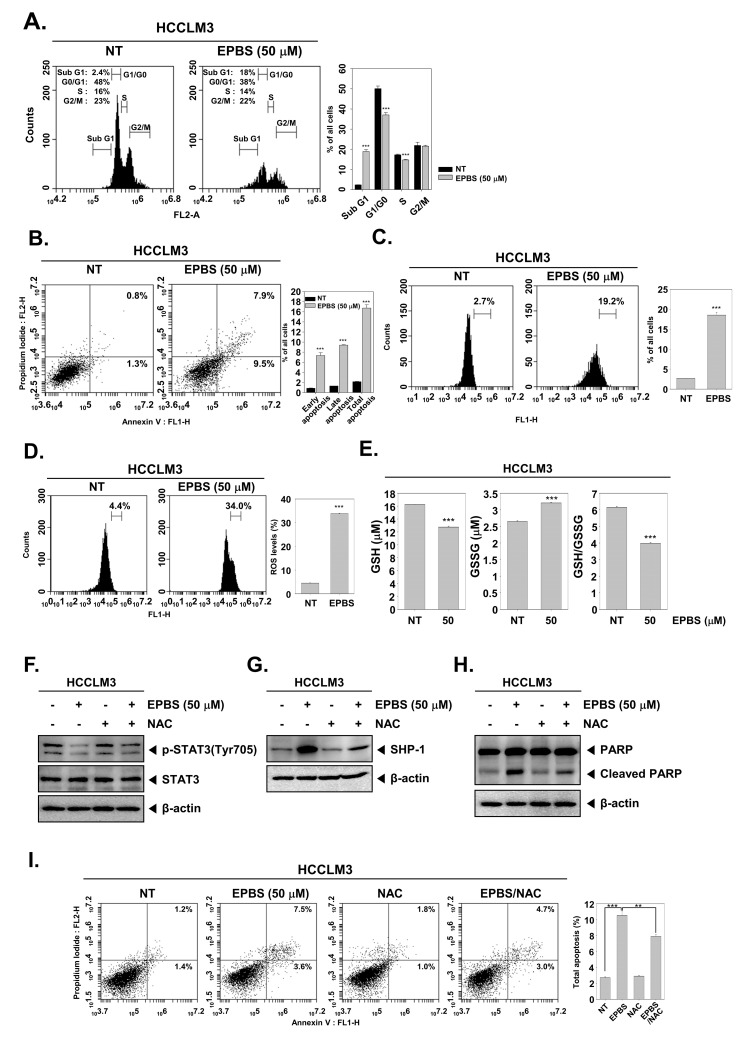
EPBS–induced cell death through ROS production. (**A**–**C**) HCCLM3 cells were treated with EPBS for 24 h and fixed EtOH. Cell cycle analysis, Annexin/PI staining analysis, and TUNEL assay were performed. *** *p* < 0.001 vs. non-treated (NT) cells. (**D**) HCCLM3 cells were treated with EPBS for 12 h, and the treated cells were reacted with H2DCFH-DA for 30 min at 37 °C. *** *p* < 0.001 vs. non-treated (NT) cells. (**E**) The cells were incubated with EPBS (50 µM) for 24 h and GSH/GSSG assay was conducted according to the manufacturer’s instructions. Luminescence was measured using luminescence readers. *** *p* < 0.001 vs. non–treated (NT) cells. (**F**,**G**) The cells were treated with 50 µM of EPBS or 3 mM of NAC for 6 h. (**H**,**I**) HCCLM3 cells were treated with EPBS or NAC (3 mM) for 24 h. Thereafter, Western blotting and Annexin/PI staining analysis were performed. *** *p* < 0.001 and ** *p* < 0.01 vs. EPBS–treated cells. Abbreviations: Euphorbiasteroid (EPBS); glutathione (GSH); N-acetyl-l-cysteine (NAC); signal transducer and activator of transcription 3 (STAT3).

According to the results shown in Figure 3F, the expression of oncogenic proteins such as Bcl-2, Bcl-xL, survivin, IAP-1, and cyclin D1 [46] were downregulated upon EPBS exposure. Also, the mRNA levels of Bcl-2 and survivin were effectively inhibited (Figure 3G). Caspase-3 and PARP are known as apoptotic proteins [47,48], and their cleaved form increased following EPBS exposure (Figure 3H). Interestingly, as displayed in Figure 3I, the knockdown of *SHP-1* by siRNA transfection could substantially decrease the apoptosis induction upon EPBS exposure. These results clearly establish the apoptotic effect of EPBS against HCC cells. In addition, EPBS induced cell cycle arrest in the Sub-G1 phase (Figure 4A). An Annexin V assay was conducted to analyze the apoptotic cells, and as shown in Figure 4B, early apoptosis and late apoptosis were induced in the presence of EPBS. Moreover, the data obtained from conducting a TUNEL assay showed the substantial increase of apoptotic cells (Figure 4C).

### 2.8. EPBS Affected ROS Production in HCC

As shown in Figure 4D, ROS levels increased following EPBS treatment. Also, a GSH/GSSG assay could act as a potential marker of oxidative stress because GSH could change oxidized glutathione (GSSG) in the presence of ROS [49]. GSH levels decreased and GSSG increased upon EPBS treatment, and the GSH/GSSG rate decreased (Figure 4E). These results demonstrated that EPBS could induce oxidative stress.

### 2.9. ROS Inhibition Reduced EPBS-Induced Apoptosis

Next, we examined whether oxidative stress could interact with STAT3 phosphorylation, SHP-1, and apoptosis. As shown in Figure 4F, p-STAT3 inhibition by EPBS reversed upon NAC treatment. Moreover, the upregulation of SHP-1 by EPBS was reduced with NAC treatment (Figure 4G). This pattern remained identical in the expression of cleaved PARP (Figure 4H). In Figure 4I, the total apoptotic cells increased after EPBS treatment, but following co-treatment with NAC, the percentage of total apoptotic cells decreased. These findings implied that EPBS induced apoptotic activation via ROS.

### 2.10. EPBS Promoted Autophagic Cell Death

**Figure 5:**
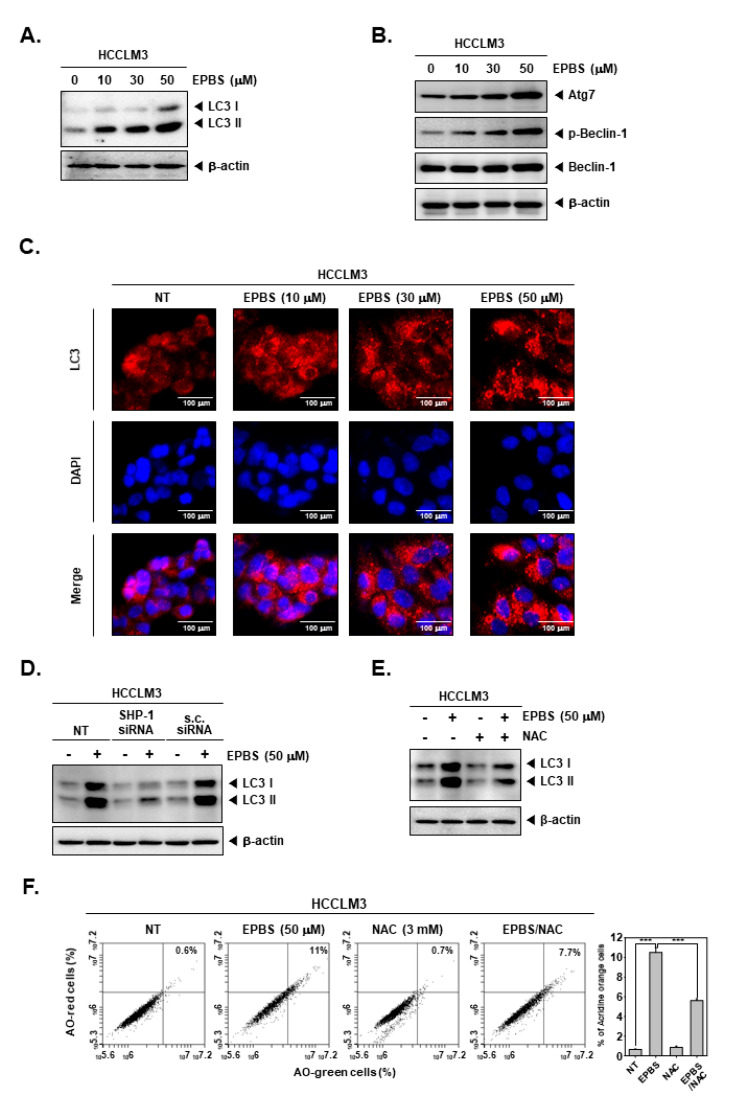
EPBS induced autophagy activations through ROS production and SHP-1. (**A**,**B**) HCCLM3 cells were treated with EPBS for 24 h and Western blot analysis was performed. (**C**) HCCLM3 cells were incubated with EPBS for 24 h. Then, immunocytochemistry was conducted for the purpose of analyzing LC3 puncta. DAPI was used for detecting nuclei. (**D**) HCCLM3 cells were transfected with *SHP-1* siRNA (50 nM) for 24 h and with EPBS for 24 h, and then Western blotting was performed. (**E**,**F**) HCCLM3 cells were treated with 50 µM of EPBS or 3 mM of NAC for 24 h, and were then collected. Thereafter, Western blot analysis and AO staining assay were performed. *** *p* < 0.001 vs. EPBS-treated cells. Abbreviations: Euphorbiasteroid (EPBS); N-acetyl-l-cysteine (NAC); scrambled siRNA (s.c. siRNA).

ROS are related to autophagy [50], and excessive autophagy could cause autophagic cell death [22]. In addition, drugs targeting apoptosis alone can cause cancer to become drug-resistant. Therefore, since it is important to target various cell death types, we investigated how EPBS affects autophagy. As displayed in Figure 5A,B, the expression of LC3, Atg7, and p-Beclin-1 was increased by EPBS treatment. When the cells are undergoing autophagy, LC3 puncta can be observed. Thus, we performed an ICC assay (Figure 5C). The puncta of LC3 was clearly detected with the increased doses of EPBS. Furthermore, we investigated whether SHP-1 could affect autophagy. In the results of Figure 5D, the *SHP-1* knockdown by siRNA transfection could affect induced LC3 levels by EPBS. It was also observed that the expression levels of LC3 were substantially suppressed upon exposure to NAC (Figure 5E). In addition, AO staining assay data showed that AO stained cells were reduced to a greater extent following their co-treatment with EPBS/NAC in comparison to those that only received EPBS treatment (Figure 5F). These results demonstrated that EPBS could induce autophagy via driving STAT3 inhibition and ROS production.

## 3. Discussion

HCC accounts for about 90% of liver cancer [23]. The abnormal expression of STAT3 is common in HCC, and this promotes cancer progression [51]. The purpose of this study was to analyze the possible anti-cancer efficacy of EPBS and to elucidate its potential impact on diverse oncogenic pathways in HCC. In HCCLM3 cells and Hep3B cells, EPBS successfully inhibited the STAT3 signaling pathway, and induced apoptosis and autophagy. We found that EPBS attenuated the IL-6-induced STAT3 activity and the expression of its upstream kinases, JAK1 and Src. In addition, we demonstrated that EPBS can induce the activation of SHP-1 protein, which can block phosphorylation of STAT3 [9], thereby inducing apoptosis and autophagy. Moreover, EPBS can downmodulate the expression of various oncogenic proteins involved in tumorigenesis.

We found that EPBS inhibited the phosphorylation of STAT3 at tyrosine 705 residue, which was associated with JAK1/Src inhibition [52,53]. In our study, we confirmed the repression of STAT3 activities, such as DNA binding or translocation into the nucleus, as a transcription factor, using EMSA and ICC assays [54]. A similar trend occurred in the STAT3 inducible Hep3B cell line.

There are several types of PTPs, such as PTP epsilon, SHP-1/2, PTEN, and CD45, which are known to negatively regulate the phosphorylation of STAT3 [55,56]. We confirmed that EPBS specifically induced SHP-1 but not SHP-2 expression. Additionally, we conducted an experiment involving the knockdown of the *SHP-1* gene by siRNA transfection to investigate whether the overall anticancer effect of EPBS was caused by SHP-1 induction. Interestingly, the knockdown of SHP-1 increased the expression of p-STAT3 [57,58] and affected the reduction of p-STAT3 expression by EPBS. We found that *SHP-1* knockdown caused changes in the expression of apoptotic proteins and autophagy-related proteins affected by EPBS. These results implied that SHP-1 can negatively regulate the STAT3 activation, and EPBS can effectively inhibit STAT3 expression through SHP-1 to induce its anti-cancer activity.

ROS has been reported to modulate both cell growth and cell death [59,60]. Since ROS is involved in many types of cell death, including apoptosis and autophagy [61,62], we confirmed the effect of ROS upon EPBS treatment. NAC is known to reduce oxidative stress [63], so we adopted it as an ROS inhibitor. The combined treatment of NAC and EPBS slightly reduced the effect of EPBS on the down-regulation of p-STAT3 expression, increasing the expression of SHP-1, inducing the expression of apoptotic proteins, and causing up-regulation of autophagy-related proteins. Thus, it was demonstrated that EPBS could inhibit STAT3 activation through the promotion of ROS generation, which can cause apoptosis as well as autophagy.

Overall, our study demonstrated for the first time that EPBS could display anticancer efficacy in HCC by potentially affecting STAT3 activation. Furthermore, it was confirmed that EPBS could cause two different forms of cell death, effects-apoptosis and autophagy, through the generation of ROS and the induction of SHP-1.

Although this study used a normal mouse liver cell line, we confirmed it had no cytotoxicity. It is hoped that more studies can advance to the clinical stage through in vivo experiments. Since STAT3 is expressed in almost all carcinomas [64,65], it is thought that the anti-cancer effect of EPBS in various carcinomas is possible, and its efficacy in various carcinomas will be revealed through future studies.

## 4. Materials and Methods

### 4.1. Reagents

Euphorbiasteroid (Catalog No. CFN90641) was obtained from ChemFaces (Wuhan, Hubei, China). EPBS was stored in dimethyl sulfoxide (DMSO) at a concentration of 50 mM, diluted in media during drug treatment, and used at the desired concentration. Dulbecco Modified Eagle Medium (DMEM) high glucose medium and fetal bovine serum (FBS) were purchased from Thermo Scientific HyClone (Waltham, Massachusetts (MA), United States (USA)). Interleukin-6 (IL-6), Alexa FluorTM 594 donkey anti-rabbit IgG (H+L) antibody, and iNfectTM in vitro Transfection Reagent was procured from Intron Biotechnology Inc. (Burling, MA, USA). Anti-p-STAT3, anti-p-JAK1, anti-JAK1, anti-p-Src, anti-Src, anti-Cyclin D1, anti-Cleaved caspase-3, anti-PARP, anti-LC3, anti-Atg7, anti-p-Beclin-1, and anti-Beclin-1 antibodies were obtained from Cell Signaling Technology (Danvers, MA, USA). Anti-STAT3, anti-β-actin, anti-SHP-1, anti-SHP-2, anti-Bcl-2, anti-Bcl-xL, anti-Survivin, anti-IAP-1, and anti-Caspase-3 antibodies were purchased from Santa Cruz Biotechnology (Dallas, TX, USA).

### 4.2. Cell Lines and Culture Conditions

HCC Hep3B cells were purchased from American Type Culture Collection (Manassas, VA, USA). HCCLM3 cells were kindly provided by Prof. Kam Man Hui, from the National Cancer Centre, Singapore. Both cells were cultured in DMEM high glucose medium containing 1% P/S and 10% inactivated FBS. NCTC Clone 1469 cells were obtained from Korea Cell Line Bank. NCTC Clone 1469 cells were cultured with DMEM high medium containing 10% horse serum and 1% P/S. Those cells were maintained at 37 °C in an incubator. The cells were sub-cultured at ~80% confluence.

### 4.3. MTT Assay

A 2,5-diphenyl-2H-tetrazolium bromide (MTT) assay was conducted to analyze the cytotoxic effect of EPBS. HCCLM3 and Hep3B cells (1 × 10^4^ cells/well) were incubated in a 96-well plate. Both types of cells were treated with 0–5–10–30–50–100 µM of EPBS for 24 h. A total of 2 mg/mL MTT solution was treated for 2 h, and an MTT lysis buffer was treated overnight in the incubator [66].

### 4.4. Western Blot Analysis

HCCLM3 and Hep3B cells were seeded on 35 π (pi) plates and incubated overnight. The cells were incubated with the indicated concentration of EPBS for the indicated time intervals. The cells were harvested and lysed, and the same protein amounts were calculated using a Bradford assay. The subsequent procedures were the same as those mentioned before [14].

### 4.5. Immunocytochemistry

The HCCLM3 cells were seeded the 8-well chamber slide (2 × 10^4^ cells/well). After EPBS treatment, the HCCLM3 cells were fixed with 4% paraformaldehyde (PFA) for 20 min and washed 3 times with PBS. An amount of 0.2% triton X-100 was treated for 10 min, and the cells were blocked with 5% BSA in PBS for 1 h. The first antibody was treated overnight at 4 °C and the second antibody was treated for 1 h. To detect the nuclei of the cells, DAPI was stained for 3 min and mounted cover glass using Fluorescent Mounting Medium (Sigma-Aldrich, St. Louis, MI, USA). The samples were detected using a FluoView FV1000 confocal microscope (Olympus, Tokyo, Japan).

### 4.6. Electrophoretic Mobility Shifts Assay (EMSA) for STAT3 Binding

The cells were seeded on 6-well plates (5 × 10^5^ cells/well) and treated with EPBS. Then, nuclear extracts were prepared and incubated with the STAT3 oligonucleotide. The DNA and protein complex form was loaded in polyacrylamide gels and transferred onto nylon membranes. Then, the samples were detected according to the manufacturer’s protocols using a LightShift^TM^ Chemiluminescent EMSA kit (Thermo Fisher Scientific Inc., Waltham, MA, USA).

### 4.7. Luciferase Assay

The Hep3B cells (8 × 10^4^ cells/well) were transfected with 300 ng of STAT3-luciferase DNA and STAT3 dominant-negative DNA using an iNfect^TM^ in vitro Transfection Reagent (Intron biotechnology Inc., Burling, MA, USA). After 48 h, the transfected cells were washed and incubated with EPBS and IL-6. The cells were collected and lysed with a lysis buffer. The luciferase assay was performed according to previously detailed procedures [67].

### 4.8. SHP-1 siRNA Transfection

The HCCLM3 cells (8 × 10^4^ cells/well) were incubated on a 24-well plate and transfected with *SHP-1* siRNA (50 nM) and scramble siRNA (50 nM) for 24 h using an iNfect^TM^ in vitro Transfection Reagent. Then, the cells were treated with 50 µM EPBS for 6 h or 24 h.

### 4.9. Reverse Transcription Polymerase Chain Reaction (RT-PCR)

To confirm the mRNA levels of *SHP-1*, the HCCLM3 cells were treated with 0–10–30–50 µM of EPBS for 6 h. To assess the levels of *Bcl-2* and *Survivin* mRNA, the cells were incubated in EPBS for 24 h. After treatment, the HCCLM3 cells were harvested, and cDNA was prepared using a Trazol reagent. GAPDH was used for a control. RT-PCR and RT-qPCR were performed according to previously detailed procedures [68].

### 4.10. Cell Cycle Analysis

The HCCLM3 cells were treated with 50 µM of EPBS for 24 h and fixed with EtOH at 4 °C overnight. The fixed cells were incubated with 1 mg/mL of RNase A for 1 h at 37 °C in an incubator and treated with propidium iodide (PI). The stained cells were detected using a BD Accuri^TM^ C6 Plus Flow Cytometer (BD Biosciences, Franklin Lakes, NJ, USA).

### 4.11. Annexin/PI Staining Assay

The HCCLM3 cells were treated with 50 µM of EPBS for 24 h, and the collected cells were stained with FITC-tagged Annexin V antibodies and PI for 10 min. The rate of apoptotic cells was analyzed using a BD Accuri^TM^ C6 Plus Flow Cytometer (BD Biosciences, Franklin Lakes, NJ, USA). 

### 4.12. TUNEL Assay

The EPBS-treated HCCLM3 cells were incubated with 4% PFA in DW for 30 min and 0.2% triton X-100 was treated for 10 min. A TUNEL staining assay was conducted according to the manufacturer’s instructions. The stained cells were detected using a BD Accuri^TM^ C6 Plus Flow Cytometer (BD Biosciences) [35].

### 4.13. ROS Measurement

The HCCLM3 cells were seeded in 35 pi plates (5 × 10^5^ cells/well). EPBS was treated for 12 h and the cells were collected. A total of 10 µM of DCFH-DA was treated for 30 min at 37 °C, and the samples were analyzed using a BD Accuri^TM^ C6 Plus Flow Cytometer (BD Biosciences) [32].

### 4.14. GSH/GSSG Assay

The GSH/GSSG assay was performed according to the manufacturer’s methods. The glutathione levels were detected using a GSH/GSSG-Glo Assay (Promega, Madison, WI, USA).

### 4.15. Statistical Analysis

All values are represented as the mean ± SD. To obtain the statistical significance, a Student’s unpaired *t*-test was performed. *** *p* < 0.001, ** *p* < 0.01, and * *p* < 0.05 vs. non-treated (NT) cells.

## 5. Conclusions

**Figure 6:**
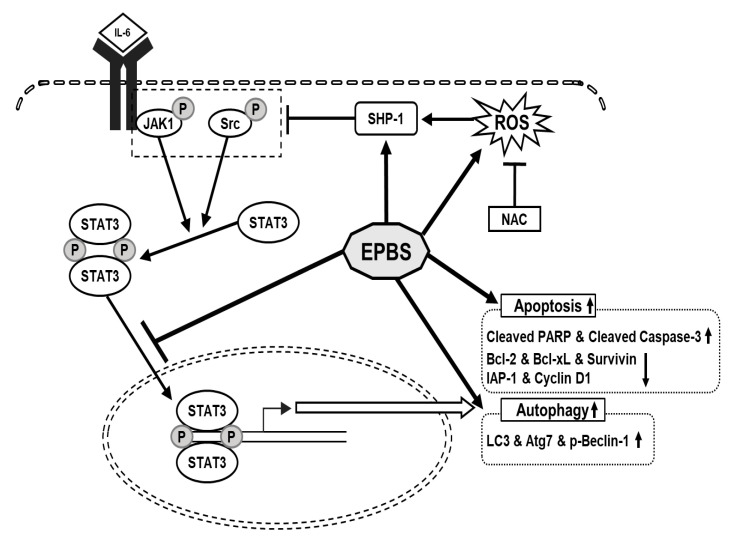
The schematic diagram of the anti-cancer effect of EPBS. EPBS induced ROS and SHP-1. Also, EPBS inhibited phosphorylation of STAT3 through inhibition of JAK1 and Src. As a result, EPBS induced apoptosis and autophagy, leading to cell death. Abbreviations: Euphorbiasteroid (EPBS); Interleukin-6 (IL-6); Janus kinase 1 (JAK1); N-acetyl-l-cysteine (NAC); reactive oxidative stress (ROS); signal transducer and activator of transcription 3 (STAT3).

We confirmed that EPBS had cytotoxic effects in HCC. We used a STAT3- constitutive cell line and a STAT3-inducible cell line for the experiment. IL-6 was used for the inducible STAT3 inducer, and it successfully reacted. EPBS stimulated ROS levels and SHP-1 expression, and those led to the down-regulation of p-JAK1 and p-Src. The p-STAT3 dimers translocated into the nucleus, but due to EPBS treatment, this activity of the transcription factor was inhibited. Apoptotic proteins and protein-related autophagy were up-regulated with EPBS treatment (Figure 6). Taken together, our findings implied that EPBS could act as an anti-cancer reagent by inducing apoptosis and autophagy.

## Data Availability

The data presented in this study are available on request from the corresponding author.
